# Expression of concern to: High systematic levels of the cytokine-inducing HMGB1 isoform secreted in severe macrophage activation syndrome

**DOI:** 10.1186/s10020-020-0142-x

**Published:** 2020-02-03

**Authors:** Karin Palmblad, Hanna Schierbeck, Erik Sundberg, Anna-Carin Horne, Helena Erlandsson Harris, Jan-Inge Henter, Daniel J. Antoine, Ulf Andersson

**Affiliations:** 1 Unit of Pediatric Rheumatology, Department of Women’s and Children’s Health, Karolinska Institutet, Karolinska University Hospital, Solna, Karolinska Institutet, Stockholm, Sweden; 2 Rheumatology Unit, Department of Medicine, Karolinska Institutet, Karolinska University Hospital, Solna, Karolinska Institutet, Stockholm, Sweden; 3 Childhood Cancer Research Unit, Department of Women’s and Children’s Health, Karolinska Institutet, Karolinska University Hospital, Solna, Karolinska Institutet, Stockholm, Sweden; 4 Medical Research Council Centre for Drug Safety Science, Department of Molecular and Clinical Pharmacology, University of Liverpool, Liverpool, UK

**Expression of Concern to: Mol Med (2014) 20:538–547.**

**https://doi.org/10.2119/molmed.2014.00183**

The Editors-in-Chief would like to alert readers that this article (Palmblad et al., 2014) is part of an investigation being conducted by the journal following the conclusions of an institutional enquiry at the University of Liverpool with respect to the quantitative mass spectrometry-generated results regarding acetylated and redox-modified HMGB1. Appropriate editorial action will be taken once the investigation is concluded.

Karin Palmblad, Anna-Carin Horne, Helena Erlandsson Harris, Jan-Inge Henter, and Ulf Andersson agree to this editorial expression of concern.

Hanna Schierbeck, Erik Sundberg, and Daniel J. Antoine have not responded to any correspondence from the editor/publisher about this editorial expression of concern.
